# Self-Assembled Verteporfin Nanoparticles for Photodynamic and Light-Independent Therapy in Glioblastoma

**DOI:** 10.1002/anbr.202400098

**Published:** 2024-09-28

**Authors:** John A. Quinlan, Kaylin Baumiller, Anandita Gaur, Wen-An Chiou, Robert W. Robey, Michael M. Gottesman, Huang-Chiao Huang

**Affiliations:** Fischell Department of Bioengineering, University of Maryland, College Park, MD 20742, USA; Laboratory of Cell Biology, Center for Cancer Research National Cancer Institute National Institutes of Health Bethesda, MD 20892, USA; Fischell Department of Bioengineering, University of Maryland, College Park, MD 20742, USA; Fischell Department of Bioengineering, University of Maryland, College Park, MD 20742, USA; Advanced Imaging and Microscopy Laboratory Maryland Nano Center, University of Maryland College Park, MD 20742, USA; Laboratory of Cell Biology, Center for Cancer Research National Cancer Institute National Institutes of Health Bethesda, MD 20892, USA; Laboratory of Cell Biology, Center for Cancer Research National Cancer Institute National Institutes of Health Bethesda, MD 20892, USA; Fischell Department of Bioengineering, University of Maryland, College Park, MD 20742, USA; Marlene and Stewart Greenebaum Comprehensive Cancer Center, University of Maryland School of Medicine, Baltimore, MD 21201, USA

**Keywords:** amorphous drug nanoparticles, glioblastomas, Hippo pathway modulations, photodynamic therapies, verteporfin

## Abstract

Verteporfin (VP) has been used for photodynamic therapy (PDT) for over 20 years, and new applications have brought it back into the spotlight. VP is hydrophobic and requires lipid carriers for clinical delivery as Visudyne. A nanosuspension of VP, termed NanoVP, that requires no carriers is developed, permitting delivery of VP alone in an aqueous solution. NanoVP is produced by solvent–antisolvent precipitation, with dimethyl sulfoxide as the preferable solvent of several screened. The initial formulation has a hydrodynamic diameter of 104 ± 6.0 nm, concentration of 133 ± 10 μM, polydispersity index (Pdi) of 0.12 ± 0.01, and zeta potential of −22.0 ± 0.93 mV. Seeking a concentration >500 μM, a zeta potential <−10 mV, a diameter <64 nm, and a Pdi < 0.2, eight synthesis parameters are probed, identifying three that modified nanoparticle diameter and three that modified nanoparticle dispersity. The diameter is tuned fourfold from 49.0 ± 4.4 to 195 ± 7.1 nm, and the solution concentration is increased by 6.3-fold to 838 ± 45.0 μM. Finally, the bioavailability and anticancer capacity of NanoVP in glioblastoma are evaluated. In all, this provides a framework for the modification of amorphous nanoparticle properties and a new formulation for clinical use of VP.

## 1. Introduction

It is estimated that ≈90% of potential new drugs are poorly water soluble,^[1]^ compared to only ≈40% of currently used pharmaceutical agents.^[2]^ This gap may indicate that hydrophobic drugs are translated to the clinic at a disproportionately low rate, potentially due to unfavorable pharmacokinetics and bioavailability. Various nanoparticle-based technologies have been developed to enhance the bioavailability of hydrophobic drugs,^[3]^ reduce toxicity,^[4]^ enable codelivery of synergistic agents,^[5]^ and target delivery of chemotherapeutics.^[6]^ Nevertheless, nanocarriers have drawbacks. For example, liposomes can potentially induce antiliposomal immunity^[7]^ thus hastening clearance, and surfactants can induce anaphylaxis and hypersensitivity.^[8]^ Still, liposomes and lipid carriers have proven clinically useful in the delivery of anticancer, antifungal, anti-inflammatory,^[9]^ and mRNA therapeutics.^[10]^ Biologically inspired delivery routes, such as extracellular vesicles^[11]^ and antibody-drug conjugates,^[12]^ are promising and in various stages of clinical translation. Successful translation of polymeric nanoparticles to the clinic is limited, potentially due to difficult-to-predict nonspecific cellular uptake and difficulties ensuring therapeutic cargo reaches the proper cellular location.^[13]^ As such, only two polymeric formulations, both of paclitaxel, have received clinical approval worldwide, and none have been approved by the Food and Drug Administration (FDA).^[14]^ While nanocarrier-based drug delivery makes intentional targeting^[15]^ as well as both active^[16]^ and passive^[17]^ accumulation of chemotherapeutics with reduced side effects^[18]^ feasible, there is still reason to pursue carrier-free solutions that do not require confounding chemicals for delivery.

Carrier-free nanosuspensions help overcome drug solubility and bioavailability issues, and the simplicity of the system improves the potential to translate the product to market, removes potential toxic side effects of excipients, and permits around 100% drug loading within particles.^[19]^ The best-studied nanodrug for anticancer effects is paclitaxel, a widely used chemotherapeutic drug, that stabilizes microtubules to induce defects in mitotic spindle assembly, chromosome segregation, and cell division. Pure-drug nanosuspensions of paclitaxel, as well as carrier-free codelivery of paclitaxel with other chemotherapies, photothermal agents, and photodynamic agents, have been developed.^[20]^ Work has been done to evaluate how synthesis parameters impact the diameter of paclitaxel nanoaggregates, finding that the concentration of paclitaxel in solvent and increased solvent-to-antisolvent ratios resulted in increased diameters.^[21]^ The most successful application of paclitaxel nanoaggregates is with albumin as Abraxane, which showed improved tumor response and decreased neutropenia compared to Taxol, which is paclitaxel delivered with the emulsifier Cremophor EL.^[22]^ Improved efficacy and reduced side effects were observed likely because tumoral accumulation was similar between the two agents despite fivefold lower plasma levels with Abraxane.^[23]^ This is likely attributable to improved paclitaxel distribution and tissue penetration when delivered as Abraxane compared to solvent-based paclitaxel.^[24]^ As a result, Abraxane is used in combination therapies for advanced breast cancer, nonsmall cell lung cancer, and pancreatic cancer.^[25]^

The porphyrin-based drug verteporfin (VP; also known as benzoporphyrin derivative monoacid ring A (BPD-MA)) has been used in the clinic for over 20 years with lipid carriers.^[26]^ VP has traditionally been used for photodynamic therapy (PDT) in wet age-related macular degeneration where VP is intravenously infused and activated in a site-specific manner using 690 nm light.^[27,28]^ VP has returned to clinical interest as a modulator of Hippo pathway signaling to modulate cancer cell behavior in glioblastoma (NCT04590664), as well as more traditional PDT applications in ocular disease (NCT05589974, NCT02321267, NCT03941587), prostate cancer (NCT03067051), pancreatic cancer (NCT06381154), and tumors obstructing airways (NCT06306638). For light-independent applications, VP was identified as a binder of transcriptional coactivator Yes-Associated Protein (YAP),^[29]^ blocking the ability of YAP to interact with transcriptional coactivator with PDZ-binding motif (TAZ).^[30]^ Ultimately, disruption of YAP-TAZ binding blocks recruitment of transcriptional enhanced associate domain (TEAD) transcription factors, inhibiting Hippo pathway-mediated transcription.^[29]^ This property of VP has recently been leveraged in glioblastoma in vitro,^[31]^ in vivo,^[32–34]^ and in the clinic^[33]^ (NCT04590664), in vitro for retinoblastoma^[35]^ and conjunctival melanoma,^[36]^ and in vivo for ovarian cancer,^[37]^ esophageal cancer,^[38]^ and pancreatic ductal adenocarcinoma.^[39]^ Given this renewed and expansive interest in VP as both a chemotherapy and as a photosensitizer, in which the drug may be administered repeatedly for multiple cycles and in larger doses (in the millimolar range) than for PDT applications, and the challenges associated with lipid-mediated drug delivery,^[7]^ a carrier-free formulation of the VP is desirable.

We recently described a new formulation of VP, termed NanoVP, which capitalizes on the self-aggregation of VP in an aqueous solution to form carrier-free nanoaggregates.^[40]^ Self-aggregation of VP in water has been noted for decades^[41]^ and has been observed in cells with high concentrations of the drug.^[42]^ Aggregates of various porphyrins have been detected and were historically described as “micelle-like” aggregates,^[43]^ but because VP was initially developed as a photosensitizer, concerns about reduced photoactivity in aqueous aggregated forms^[44]^ led to the clinical utility of the drug with lipids as Visudyne. Visudyne has been reported to have large and variable diameters and a high Pdi (733.8 488.9 nm, Pdi 0.66 0.20^[45]^; 524.8 nm, Pdi 0.877^[46]^). It is thought that a particle diameter <150 nm is needed to extravasate from leaky vasculature at the site of a tumor, and a diameter <50 nm helps evade mononuclear phagocyte system-mediated clearance or to permit adequate delivery to the brain.^[47]^ While Visudyne’s broad size is desirable for keeping VP in vasculature for light-mediated vascular shutdown,^[27]^ new formulations that improve delivery to tumors may be useful. Originally, liposomal VP was adopted as the delivery route for VP because it outperformed VP delivered in DMSO in early biodistribution and efficacy studies.^[48,49]^ It was noted in these studies, however, that delivery of VP in DMSO resulted in some aggregation and was not translationally relevant.^[49]^ It is now thought that VP almost immediately dissociates from lipids in Visudyne to redistribute to biomolecules in the plasma.^[50]^ Considering the practical limitations of acquiring Visudyne,^[51]^ a new focus on VP as an anticancer drug in the absence of light, and historic motivations for utilizing lipids to deliver VP, we believe NanoVP may have clinical utility. Here, we have carefully evaluated key parameters in its synthesis and stability and further evaluated its antitumor efficacy in glioblastoma.

After initially describing the capabilities of NanoVP,^[40]^ we revisited its synthesis process with new criteria. We required a concentration of at least 500 μM, which would permit intraperitoneal (IP) dosing in mice at the dosage required for light-independent use of VP. We required a diameter less than 64 nm to allow for the free diffusion of particles between cells in the brain, as others have reported that ≈75% of pores in the human brain are less than 125 nm^[52]^ and the upper limit for free diffusion is 64 nm.^[53]^ We required a Pdi < 0.2, indicating more uniform particles that may release drugs in more predictable manners.^[54]^ We desired, but did not require, a zeta potential <−30 mV. A zeta potential >+10 mV or < −10 mV is thought to prevent aggregation and particles with zeta potentials >+30 mV or <−30 mV are generally considered stable, and nega tively charged particles are considered to have less toxicity.^[55]^ Because we previously found that NanoVP dissociates in serum even with a zeta potential of −30 mV,^[40]^ a very negative zeta potential is desired but not required. Visudyne, the current clinically used formulation of VP, offers a theoretical VP concentration of 2780 μM calculated before filtration. Filtration is expected to reduce this concentration by an unknown amount as large VP-containing particles are removed from the preparation in the clinic. Because of Visudyne’s diameter (>500 nm),^[45,46]^ free diffusion in the brain is unlikely if not impossible. Additionally, Visudyne’s Pdi has been reported to be >0.7,^[46]^ which is accepted as an indicator of highly variable particle size. Visudyne has been reported to have a zeta potential of 0.155 0.210 mV^[46]^ and 0.16 0.21 mV.^[45]^ These parameters are summarized in Table 1.

We evaluate solvent–antisolvent precipitation as a method to formulate NanoVP. In this nanoparticle synthesis method, a hydrophobic material solubilized in organic solvent is mixed with an aqueous solution. This creates local supersaturation, leading to nucleation and growth of particles.^[56–58]^ We hypothesized that altering synthesis parameters in the solvent–antisolvent precipitation method would alter the dispersion of supersaturated VP in water, consequently altering the nucleation and growth rates of NanoVP particles, ultimately permitting modulation of nanoparticle diameter. We found that, of commonly available organic solvents, dimethyl sulfoxide (DMSO) permits consistently monodisperse nanoparticle formulation. Under our original synthesis conditions, we formulated nanoparticles with a diameter of 104 ± 6.0 nm and demonstrated that, through modulation of several synthesis parameters, we can tune nanoparticle size from 49.0 ± 4.4 to 195 ± 7.1 nm. We found that our nanoparticles are negatively charged and that the charge correlates indirectly with diameter. After evaluating several solutions for the delivery of NanoVP, we determined that the pH of the solution impacts the stability of the nanoparticles. We also established a pump-based system to produce NanoVP in lieu of handmade samples. In an orthotopic, patient-derived xenograft mouse model of glioblastoma (GBM), we found that VP has limited bioavailability in the brain but confirmed some anti-proliferative and antimigratory effects of NanoVP in vitro in two GBM cell lines. In all, our new NanoVP formulation presents an attractive delivery system for use in the application of VP as a chemotherapy.

## 2. Results

### 2.1. Evaluation of Solvents in Production of NanoVP

**Figure 1.**
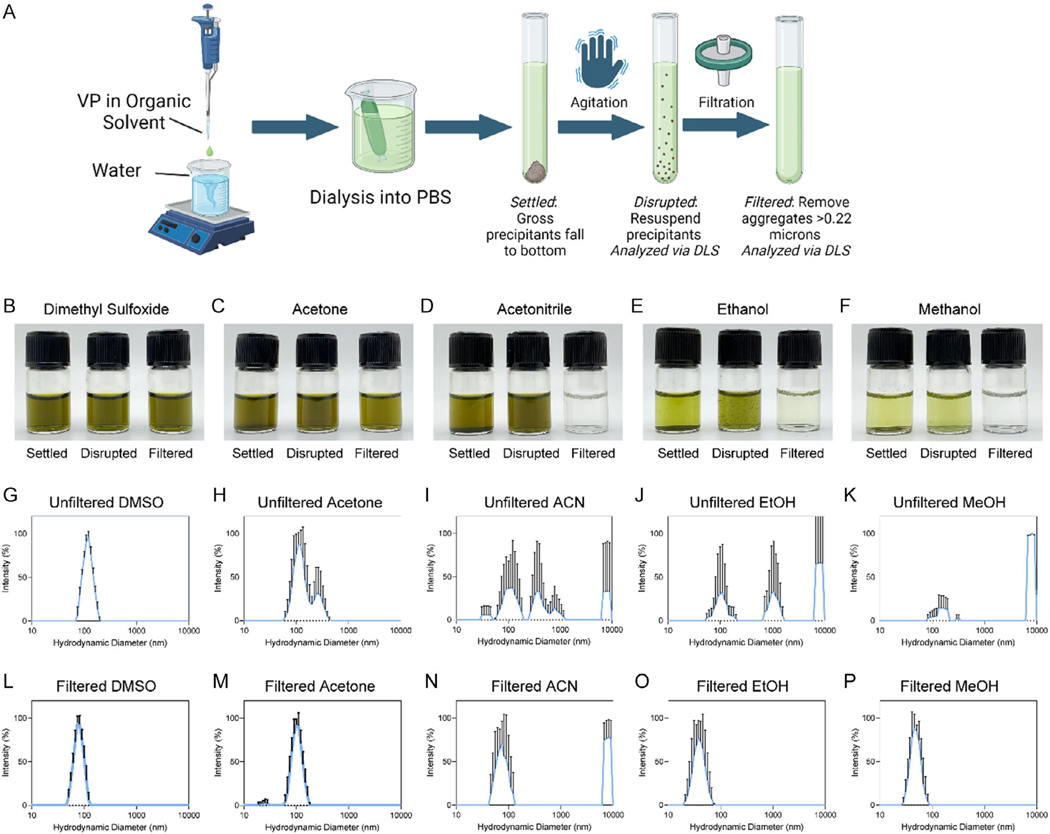
Evaluation of solvent on initial formation of nanoaggregates of VP. A) Workflow for evaluation of other solvents in the formulation of NanoVP. After dropwise solvent–antisolvent precipitation, samples were dialyzed into PBS and then allowed to settle for at least 48 h. Samples were then agitated and filtered before DLS measurements. Some samples had aggregates settle after production (left, B–F), which were manually agitated before DLS measurements (middle, B–F). Samples were also filtered before DLS measurements to remove larger aggregates (right, B–F). DLS measurements of G–K) agitated, unfiltered samples revealed larger aggregates in some cases. DLS measurements on L–P) filtered samples resulted in purified populations of nanoaggregates. Data shown as mean ± standard deviation. For all groups, *n* ≥ 3; DLS traces are from one representative sample per group, with three reads per sample. Graphic (A) made with BioRender.

Solvent–antisolvent precipitation requires the solvent for the drug of interest to be miscible with an aqueous solution, resulting in supersaturation, nucleation, and particle growth.^[59]^ We screened a panel of five organic solvents by adding VP dissolved in solvent dropwise to water, then dialyzing the suspension into phosphate-buffered saline (PBS) (Figure 1A). VP was dissolved in organic solvent at 7 mM for DMSO, acetone, and acetonitrile (ACN), and at the maximum solubility in ethanol (EtOH) (≈5 mM) and MeOH (≈4 mM). After allowing the solutions to settle, in some cases, larger aggregates and precipitates fell to the bottom of the tube (visible as dark material at the bottom of each sample; left sample of Figure 1C–F). Only DMSO did not result in the settling of gross aggregates (left sample of Figure 1B). Agitation of the sample resuspended the settled aggregates in all samples but DMSO (middle sample of Figure 1B–F). Through dynamic light scattering (DLS), we evaluated the size of particles in an agitated sample (Figure 1G–K) and in the sample filtered with a 0.22 μm filter (Figure 1L–P). Only DMSO resulted in monodisperse particles before filtration, and samples for acetone, EtOH, and MeOH resulted in single peaks after filtration. Significant material loss was noted after filtration for ACN, EtOH, and MeOH (right sample in Figure 1D–F).

### 2.2. Characterization of NanoVP under Standard Formulation Parameters

**Figure 2.**
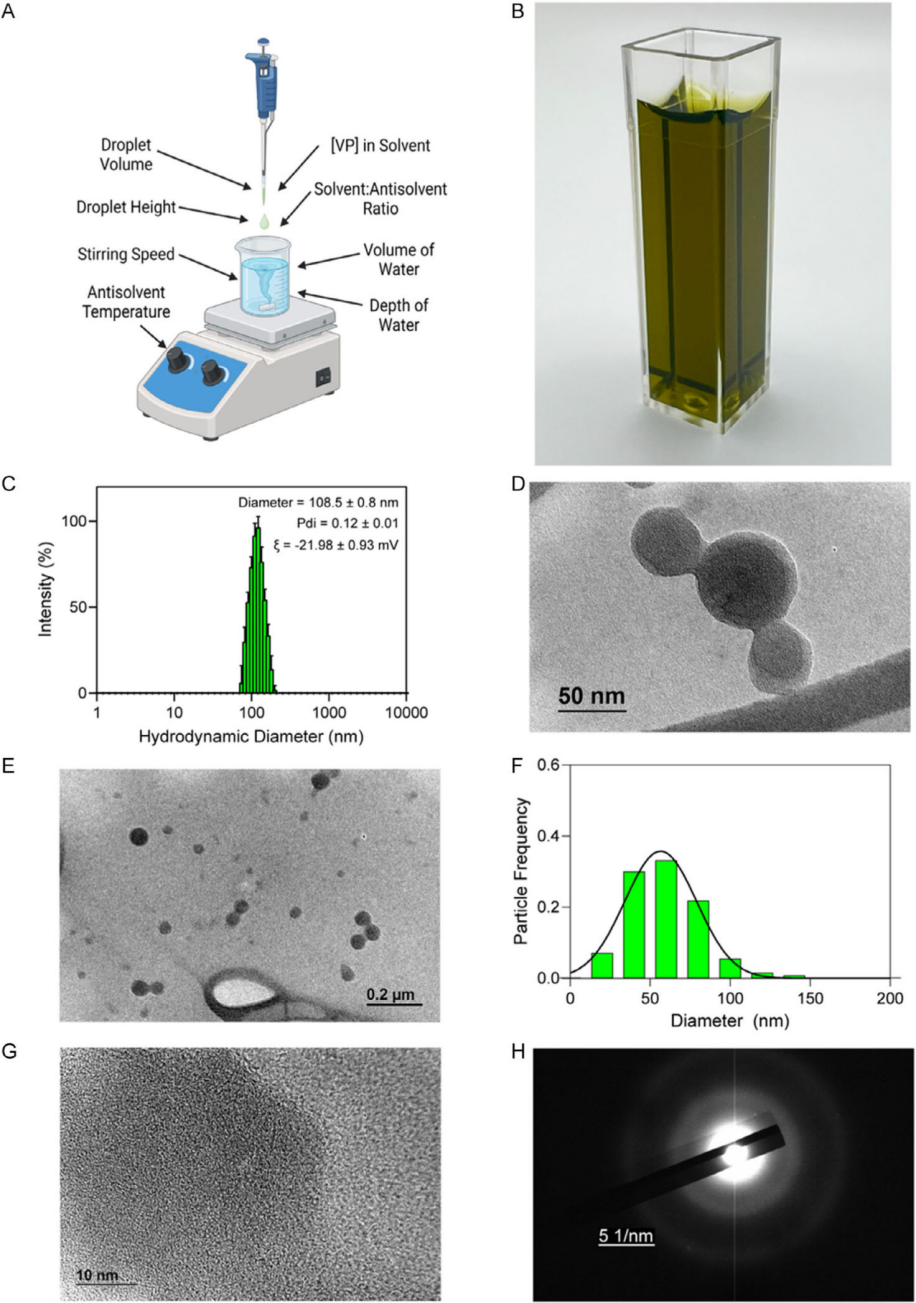
A NanoVP is formulated by solvent–antisolvent precipitation. A) VP dissolved in DMSO is added dropwise to stirring water, resulting in B) a nanosuspension that is transparent and green in color with no gross aggregates. C) NanoVP is a monodisperse with a negative charge. D,E) TEM images reveal that NanoVP is roughly spherical. F) Quantification of diameter from TEM images shows a diameter of 55.1 ± 29 nm (*n* = 380 particles). G) Closeup TEM and H) a lack of patterning in electron diffraction indicate an amorphous structure of NanoVP (scale bar is 5 1 nm^−1^). Data represented as mean ± standard deviation except for TEM quantification, where raw particle frequency is shown. For all groups, *n* ≥ 3. Graphic (A) made with BioRender.

NanoVP was formulated by solvent–antisolvent precipitation (Figure 2A) under parameters outlined in Table 1 as Condition 2. The resulting solution is a dark green translucent NanoVP (Figure 2B). NanoVP under these initial formulation conditions has a diameter of 109 ± 0.8 nm by DLS with a Pdi of 0.12 ± 0.01 and a zeta potential of −22.0 ± 0.93 mV (Figure 2C). TEM images reveal a roughly spherical particle (Figure 2D,E) with a diameter of 55.1± 29 nm (*n* = 380 particles) (Figure 2F). Close-up TEM (Figure 2G) and electron diffraction (Figure 2H) indicate particles are amorphous, with some potential crystalline structure as indicated by the faint halo, although this may be an artifact of PBS that was dialyzed off before electron diffraction or attributable to the supporting carbon film.

**Figure 3.**
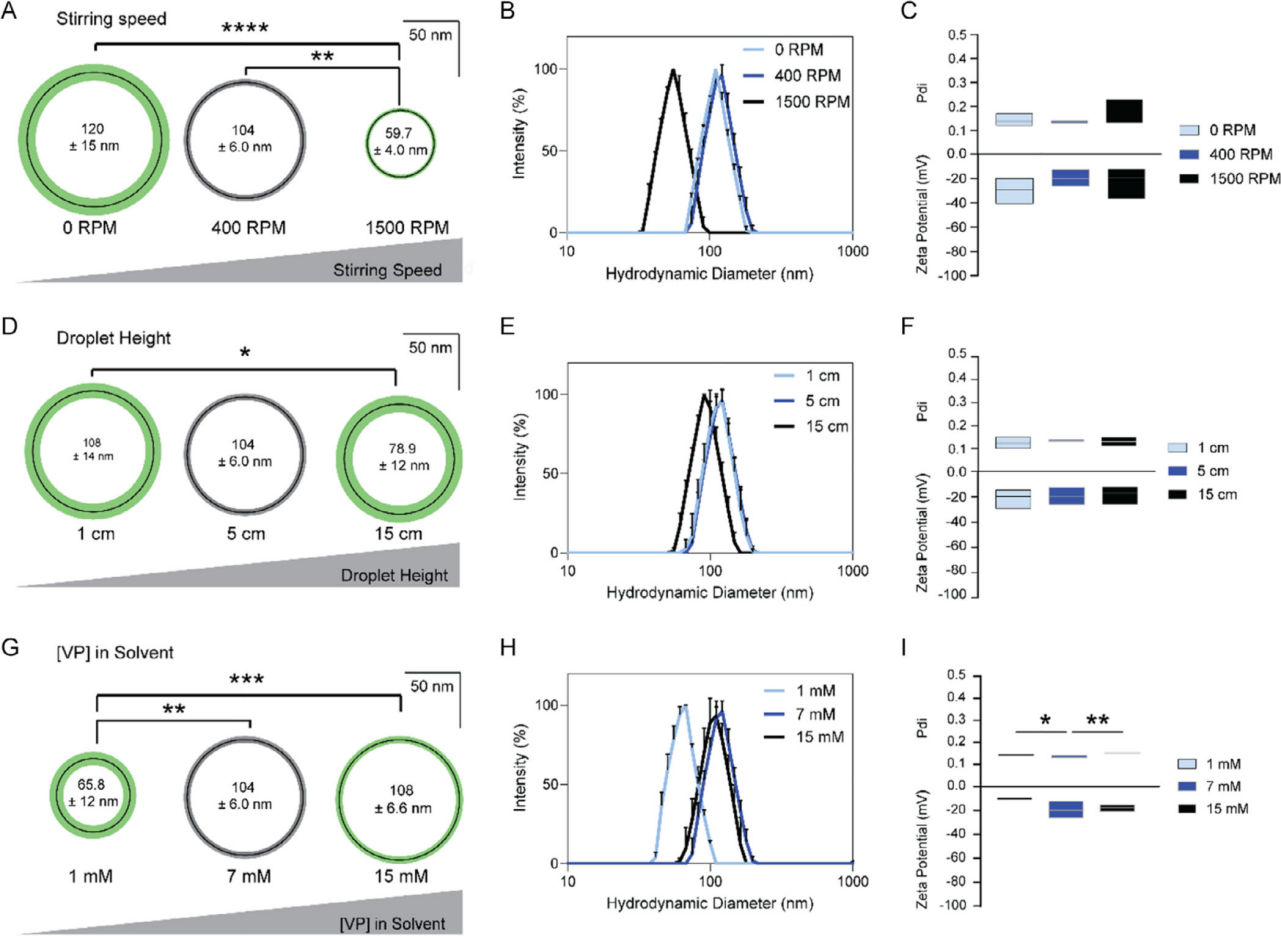
Key factors that impact the size of NanoVP. A) NanoVP decreases in diameter when stirring speed is increased, but B) remains monodisperse with C) no changes to Pdi or zeta potential. D) As the height from which VP in DMSO is dropped increases, the diameter of NanoVP decreases, maintaining a E) monodisperse profile with F) consistent Pdi and zeta potential. G) Increasing the VP concentration in DMSO increases the size of VP, resulting in H) a monodisperse population with I) a slight increase in Pdi at either extreme of concentration and no changes in zeta potential. For (A,D,G), thin black circles indicate mean diameter, and green or gray concentric rings indicate standard deviation. Gray rings indicate a representative population of NanoVP as described in Figure 1, against which all variations were compared. DLS traces show three replicate measures of a single representative sample. Pdi and zeta potential indicate the median value, with boxes extending to maximum and minimum values. For all groups, *n* ≥ 3. Statistics were determined by one-way ANOVA with Tukey’s multiple comparisons test. *p < 0.05, **p < 0.01, ***p < 0.001, ****p < 0.0001.

**Figure 4.**
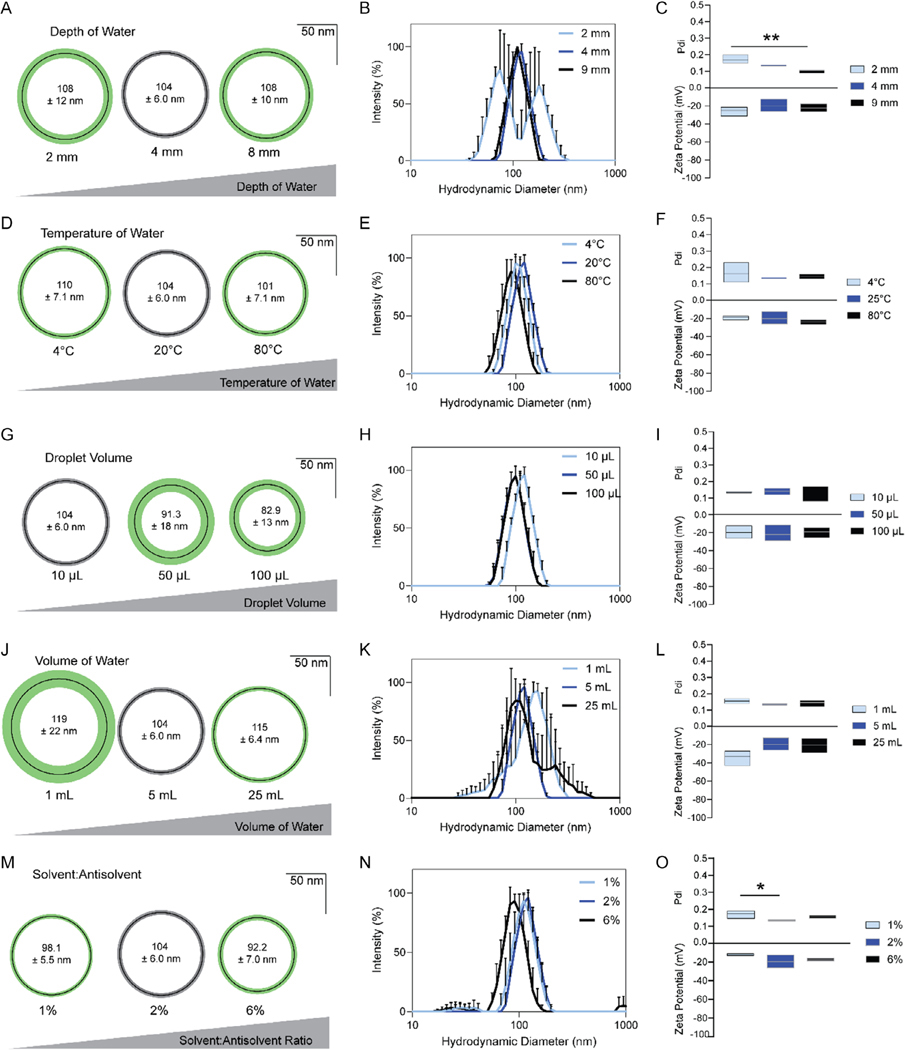
Several factors did not alter particle size but did change dispersity or zeta potential. A) Altering the depth of water while maintaining volume did not change particle size, but B) shallower water resulted in a polydisperse population with C) higher Pdi but no change to zeta potential. D,G) Temperature of water and droplet volume did not impact diameter, E,H) dispersity, or F,I) the Pdi or zeta potential of NanoVP. J) volume of water did not alter size, but K) did result in more polydisperse populations of nanoparticles at lower and higher volumes of water. L) Volume of water did not impact Pdi or zeta potential. M) Solvent-to-antisolvent ratio did not impact diameter in the tested range, with N) minimal impact on dispersity. O) Lower solvent-to-antisolvent ratios did result in a higher Pdi. For (A,D,G,J,M), thin black circles indicate mean diameter, and green or gray concentric rings indicate standard deviation. Gray rings indicate a representative population of NanoVP, as described in Figure 1, against which all variations were compared. DLS traces show three replicate measures of a single representative sample. Pdi and zeta potential indicate the median value, with boxes extending to maximum and minimum values. For all groups, *n* ≥ 3. Statistics were determined by one-way ANOVA with Tukey’s multiple comparisons test. *p < 0.05, **p < 0.01.

From initial parameters (Condition 2), we isolated individual parameters and tested a range of values one by one to evaluate impact on diameter, Pdi, and zeta potential. We combined factors based on trends to minimize size, maximize size, and then maintain concentration but reduce diameter and polydispersity of NanoVP. The “Tested Range” column indicates values tested in Figure 3 and 4. Only values that altered diameter, concentration, Pdi, or zeta potential were combined to create Conditions 1, 3, and 4. As such, certain parameters that made no impact on important nanoparticle features, such as droplet volume and volume of water, were not altered in the final combined conditions. “Design Criteria” presents parameters for the ideal formulation of NanoVP. Traits of Visudyne as reported by others^[45,46]^ are reported in Column 8. By these measures, Condition 4 best met our design goal in all traits except encapsulation efficiency and zeta potential. For measurements, data is presented as mean ± standard deviation.

### 2.3. Factors Impacting the Size, Polydispersity, or Zeta Potential of NanoVP

Of the eight identified tunable factors, we found three that significantly impacted the size of NanoVP. Stirring speed of the water was inversely proportional to diameter, as still water (0 RPM) resulted in NanoVP with a diameter of 120 ± 15 nm and water stirred at 1500 RPM resulted in NanoVP with a diameter of 59.7 ± 4.0 nm (Figure 3A). This resulted in uniform, single-peaked samples (Figure 3B) and did not impact Pdi or zeta potential (Figure 3C). The height at which the pipette was held above the surface of the water also had an inverse relationship with particle diameter. A droplet height of 1 cm resulted in NanoVP with a diameter of 108 ± 14 nm, while a droplet height of 15 cm resulted in a NanoVP diameter of 78.9 ± 12 nm (Figure 3D). Droplet height had no impact on the peakedness of DLS measurements (Figure 3E) Pdi, or zeta potential (Figure 3F). The concentration of VP in DMSO (VP in solvent) was directly related to NanoVP diameter. One millimolar (1 mM) VP in DMSO resulted in particles with a diameter of 65.8 ± 12 nm, while 15 mM VP in DMSO resulted in particles with a diameter of 108 ± 6.6 nm (Figure 3G). All concentrations of VP in DMSO resulted in uniform peaks at all concentrations tested (Figure 3H). Pdi was significantly lower with 7 mM VP in DMSO than 1 or 15 mM VP in DMSO, and there was no significant impact on zeta potential (Figure 3I).

We identified five additional factors that did not impact the diameter of the nanoaggregate. The depth of the water that VP in DMSO was dropped into did not impact size (Figure 4A), but the shallowest water resulted in split peaks on DLS (Figure 4B). The shallowest water had a significantly higher Pdi than the deepest water, with no impact on zeta potential (Figure 4C). The temperature of water and droplet volume had no impact on diameter (Figure 4D,G), peakedness (Figure 4E,H), Pdi, or zeta potential (Figure 4F,I). Volume of water, with depth of water fixed by using vessels with different diameters, had no impact on particle diameter (Figure 4J), Pdi, or zeta potential (Figure 4L), but, interestingly, smaller volumes of water or larger volumes of water led to broad peaks on DLS (Figure 4K). Finally, solvent-to-antisolvent ratio had no impact on particle diameter (Figure 4M) or peakedness (Figure 4N) or zeta potential, but Pdi was slightly lower with higher percents of DMSO in water (Figure 4O).

### 2.4. Combining Traits That Impact Nanoparticle Parameters

**Figure 5.**
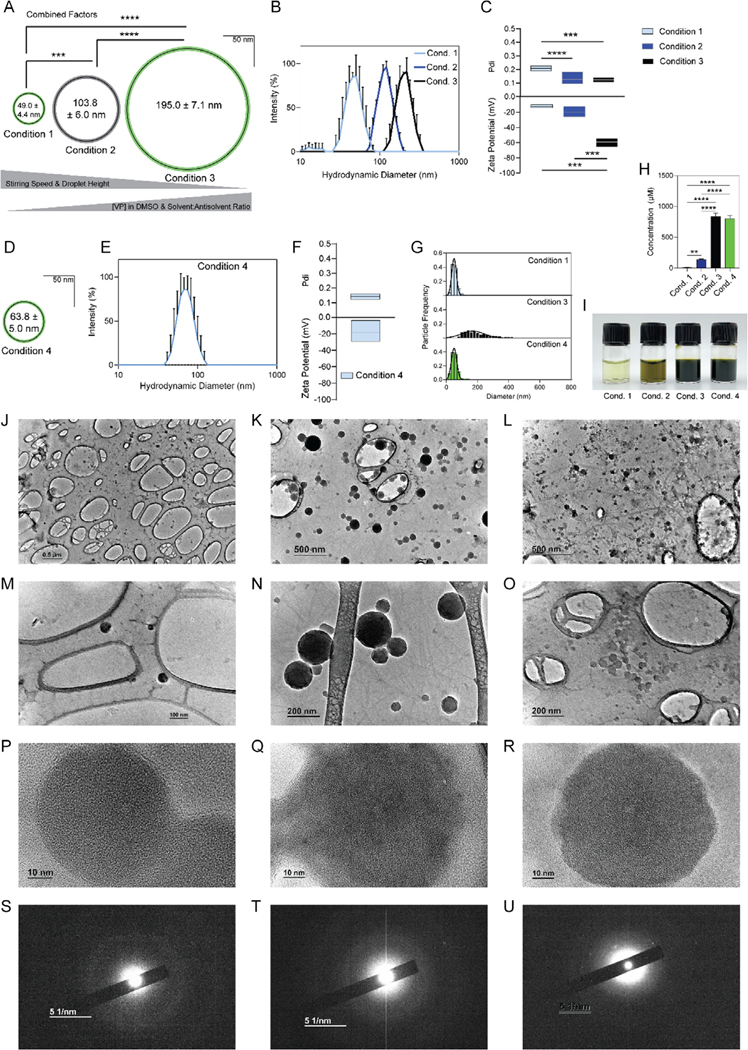
Extended range of NanoVP diameters. A) As stirring speed and droplet height decrease and VP in DMSO concentration and solvent-to-antisolvent ratio increase, the diameter of nanoparticles increases. B) This results in single-peaked samples with C) decreasing Pdi and more negative zeta potential. D) Increasing stirring speed alone decreased nanoparticle diameter, maintaining E) a monodisperse profile with F) Pdi and zeta potential around that of similarly sized nanoparticles. G) TEM image diameter quantification revealed that Conditions 1 and 4 had a monodisperse profile, while Condition 3 had a broader range of diameters. H) Concentration was significantly increased in Conditions 3 and 4. I) Intensity of green tint in the nanosuspensions (Conditions 1, 2, 3, and 4; left to right) correlates with concentration value. Wide-field TEM images for the J,M) Condition 1, K, N) Condition 3, and L,O) Condition 4 and closer-field images for P) Condition 1, Q) Condition 3, and R) Condition 4 particles show roughly spherical aggregates with a range in diameter. Very close-up images and electron diffraction of P,S) Condition 1, Q,T) Condition 3, and R,U) Condition 4 indicate that the particles remain amorphous at all diameters. In (U), scale bar is 5 1 nm^−1^. For (A, D), thin black circles indicate mean diameter, and green and gray concentric rings indicate standard deviation. Gray concentric rings indicate a representative population of NanoVP as described in Figure 1, against which all variations were compared. DLS traces show three replicate measures of a single representative sample. Pdi and zeta potential indicate the median value, with boxes extending to maximum and minimum values. For TEM quantification, raw particle frequency is shown. For all groups, *n* ≥ 3. Statistics were determined by one-way ANOVA with Tukey’s multiple comparisons test. ***p < 0.001, ****p < 0.0001.

We hypothesized that combining factors impacting nanoparticle diameter and polydispersity would enable the synthesis of particles with a greater range of diameters. As such, we altered stirring speed, droplet height, and VP concentration in solvent to modify diameter, depth of water, and solvent-to-antisolvent ratio to modify Pdi as described in Table 1. We found that these combined factors significantly increased diameter. From the Condition 2 formulation size of 104 ± 6.0 nm, combining key factors to decrease diameter resulted in Condition 1 (Table 1) nanoparticles with a diameter of 49.0 ± 4.4 nm, and combining key factors to increase diameter resulted in Condition 3 (Table 1) nanoparticles with a diameter of 195 ± 7.1 nm (Figure 5A). On DLS, all samples had similar peakedness, although the Condition 1 nanoparticles had a population of smaller nanoparticles (Figure 5B). Consistent with this, the Condition 1 nanoparticles had a higher Pdi than the Condition 2 or Condition 3 nanoparticles (Figure 5C). Interestingly, the Condition 3 nanoparticles had a significantly more negative zeta potential than the Condition 1 or Condition 2 nanoparticles (Figure 5C). Because both the solvent-to-antisolvent ratio and VP concentration in solvent were increased to make the Condition 3 nanoparticle, the resulting concentration of the nanosuspension was elevated. Desiring a more concentrated, smaller diameter particle, we increased stirring speed, which had the greatest single-factor impact on diameter, resulting in Condition 4 (Table 1, Figure 3A). The resulting nanoparticles had a diameter of 63.8 ± 5.0 nm (Figure 5D) and had a clean single peak on DLS (Figure 5E), a Pdi of 0.14 ± 0.01, and a zeta potential of −17.8 8.0 mV (Figure 5F). Diameter quantification from TEM confirmed that Condition 4 nanoparticles had a diameter of 56.2 ± 16 nm with a narrow distribution, Condition 3 nanoparticles had a diameter of 112 ± 51 nm with a broad distribution, and Condition 1 nanoparticles had a diameter of 51.8 ± 19 nm with a narrow distribution (Figure 5G). Modulating both percent solvent in antisolvent and concentration of VP in solvent permitted tunability of concentration from 9.1 ± 5.2 μM for the Condition 1 particles, 136 ± 10 μM for Condition 2 nanoparticles, 838 ± 45 μM for Condition 3 nanoparticles, and 799 ± 44 μM for Condition 4 nanoparticles (Figure 5H). By visual inspection, concentration differences are appreciable (Figure 5I, from left to right, Condition 1, Condition 2, Condition 3, and Condition 4). These different formulations resulted in an encapsulation efficiency of 92.4 ± 5.2% for Condition 1, 97.3 ± 7.1% for Condition 2, 93.1 ± 5.0% for Condition 3, and 88.7 ± 4.9% for Condition 4.

TEM confirmed that Condition 1 nanoparticles (Figure 5J,M), Condition 3 nanoparticles (Figure 5K,N), and Condition 4 nanoparticles (Figure 5L,O) all retained a roughly spherical shape. Close-up TEM and electron diffraction for Condition 1 (Figure 5P,S), Condition 3 (Figure 5Q,T), and Condition 4 (Figure 5R,U) nanoparticles indicate that the particles remain amorphous under all formulation parameters. Slight halos on electron diffraction may be attributable to residual PBS from initial dialysis or the supporting carbon film.

By the design goals outlined in Table 1, Condition 4 best met our requirements with a concentration >500 μM (799 ± 44 μM), a diameter <64 nm (63.8 ± 5.0 nm), and a Pdi < 0.20 (0.14 ± 0.01). While the zeta potential was not <−30 mV, the zeta potential of −17.8 ± 8.0 mV is below −10 mV, which should confer some stability and reduce toxicity. Additionally, the encapsulation efficiency was <90% (88.7 ± 4.9%) but is still high enough to avoid significant drug loss during preparation.

### 2.5. Relationship Between Particle Parameters

**Figure 6.**
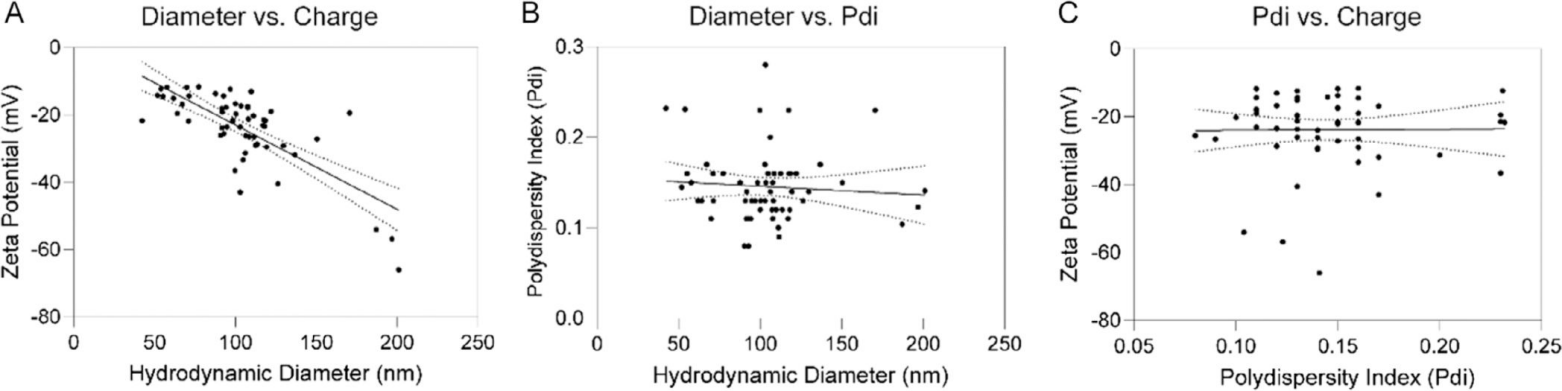
Diameter and charge are inversely related, while other nanoparticle parameters show no relationship. A) As diameter increases, zeta potential is more negative for NanoVP particles (*Y* = −0.2506 * *X* + 2.090, *R*^2^ = 0.55, *p* < 0.0001). B) Diameter versus Pdi (*Y* = −0.00009685 * *X* + 0.1555, *R*^2^ = 0.006, *p* = 0.54) and C) Pdi versus charge (*Y* = −0.001109 * *X* + 0.1176, *R*^2^ = 0.06, *p* = 0.08) show no relationship.

After observing a decrease in zeta potential in our largest nanoparticles (Figure 5C), we investigated more global trends in parameters by diameter, Pdi, and zeta potential data across many different formulation parameters. We found that diameter was indirectly related to zeta potential (Figure 6A), while there was no relationship between diameter and Pdi or Pdi and zeta potential (Figure 6B,C).

### 2.6. Impact of Carrier Solution on Stability

**Figure 7.**
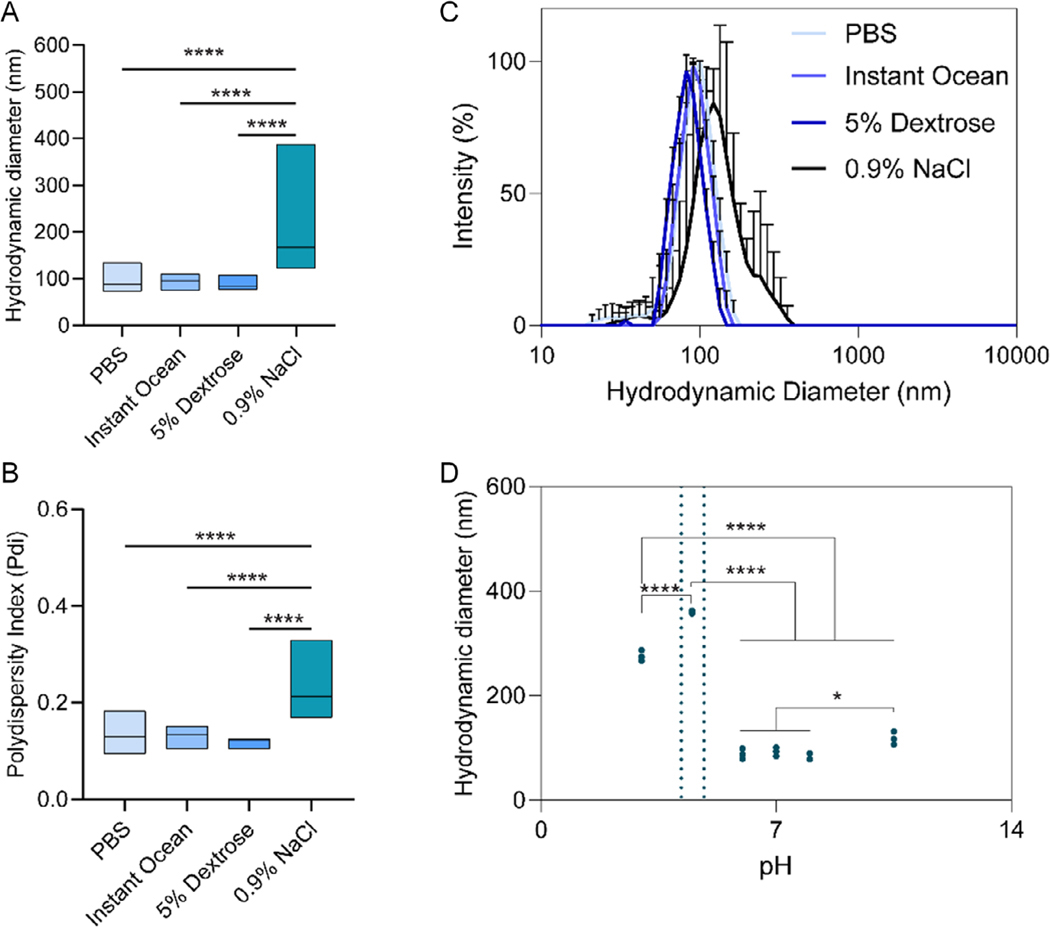
NanoVP dialyzed into different media impacts its stability. A,B) The diameter and Pdi of NanoVP remain constant in PBS, Instant Ocean, and 5% dextrose, but are lost in 0.9% sodium chloride. C) DLS reveals monodisperse particles in PBS, Instant Ocean, and 5% dextrose, but not 0.9% NaCl. D) NanoVP, when mixed with solutions of various pHs, remains stable in the pH 6–8 range, but loses stability at a pH of 10.5 and at a pH of 4.5 or 3. Light blue vertical lines in (D) indicate the acid dissociation constants (pKas) of verteporfin. For hydrodynamic diameter and Pdi, data are represented as a mean (central bar) with boxes extending to the maximum and minimum. For all groups, *n* ≥ 3. Statistics were determined by one-way ANOVA with Tukey’s multiple comparisons test. *p < 0.05, ****p < 0.0001.

Having previously found that NanoVP is stable for over 1 year in water or PBS above-freezing temperatures,^[40]^ we aimed to explore the stability of NanoVP in other solutions suitable for clinical infusion. Anticipating the use of NanoVP in experimental models and clinical settings, we evaluated the stability of NanoVP in different media. After NanoVP (Condition 2) was made in water, it was dialyzed into PBS, Instant Ocean, 5% dextrose, or 0.9% NaCl. We found that the diameter of NanoVP was unchanged in PBS, Instant Ocean, and 5% dextrose, but increased with 0.9% NaCl (Figure 7A). Similarly, the Pdi increased for 0.9% NaCl only (Figure 7B). DLS revealed that the change in size and polydispersity were likely due to a more varied population of nanoaggregates in 0.9% NaCl (Figure 7C). We probed the pH of each solution. PBS had a pH of 7.41 ± 0.04, Instant Ocean had a pH of 9.57 ± 0.09, 5% dextrose had a pH of 7.40 ± 0.20, and 0.9% NaCl had a pH of 6.42 ± 0.01. When the pH of PBS was changed and then mixed with VP, we found that stability was lost at a pH of 4.5 or lower and 10.5 (Figure 7D, light blue lines indicate the acid dissociation constants, pKas, of VP).

### 2.7. Automated Production of NanoVP

**Figure 8.**
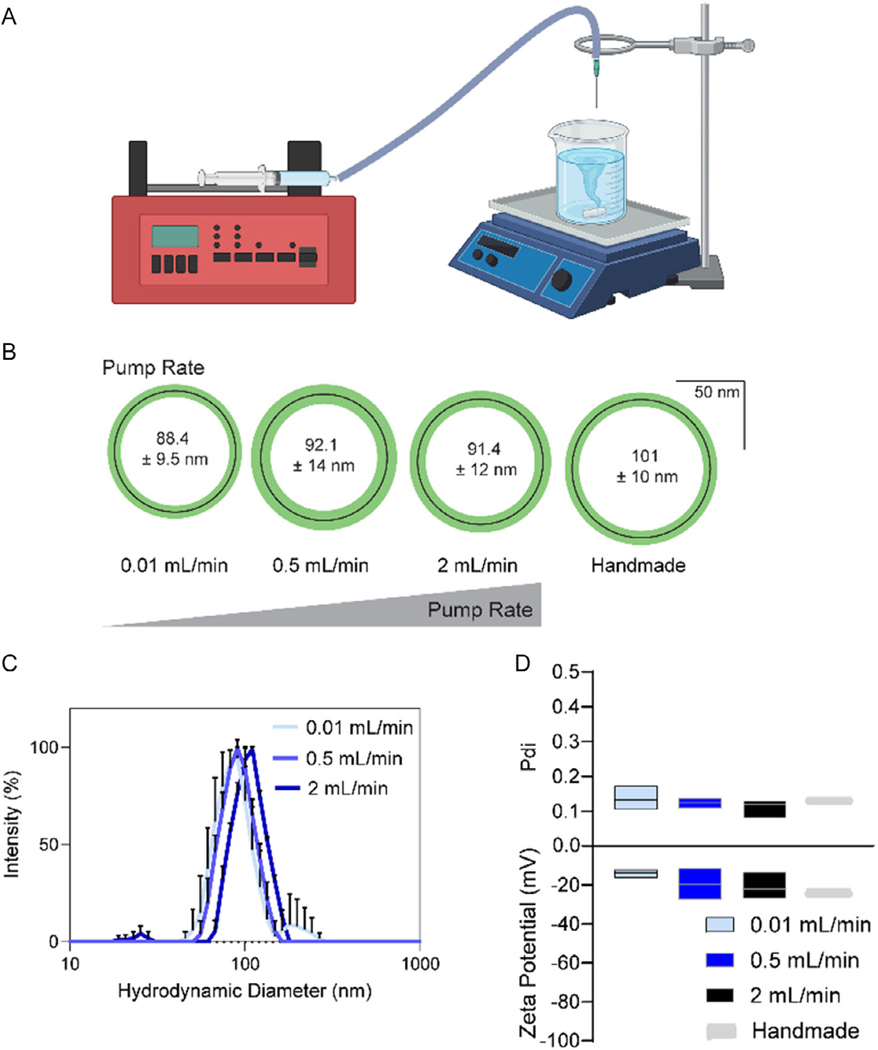
Automated production of NanoVP. A) A syringe-pump system was affixed to tubing connected to a blunt-tip needle, permitting dropwise addition of VP in DMSO to stirred water. B) With all parameters identified in handmade NanoVP held constant, altering the pump rate had no impact on size compared to handmade NanoVP. C) Pump-made NanoVP resulted in single-peaked DLS measures and D) had no impact on Pdi or zeta potential. For Pdi and zeta potential, data are represented as a mean (central bar) with boxes extending to the maximum and minimum. For all groups, *n* ≥ 3. Statistics determined by one-way ANOVA. No post-hoc test was required. Graphic (A) made with BioRender.

Aiming to improve consistency of NanoVP, we affixed a syringe pump and blunt-tip needle to either end of tubing using Luer-lock adapters (Figure 8A). We drew VP in DMSO up into the needle using the pump, thus only loading the volume of VP in DMSO needed for a single batch. NanoVP was made under the parameters listed in Condition 2 (Table 1). To evaluate the impact on droplet rate, we set the pump speed at 0.1, 0.5, or 2 mL min^−1^. We found that changing pump rate did not impact particle diameter (Figure 8B), did not result in multipeaked DLS measurements (Figure 8C), and did not impact Pdi or zeta potential (Figure 8D); all values were like that of handmade NanoVP (Figure 8B–D). It should be noted that all speeds, and the handmade process, allow the VP in DMSO to accumulate on the tip of the pipette or needle and then fall by the force of gravity. The rate at which drops fell is summarized in Table S1, Supporting Information.

### 2.8. Delivery of NanoVP to the Brain

**Figure 9.**
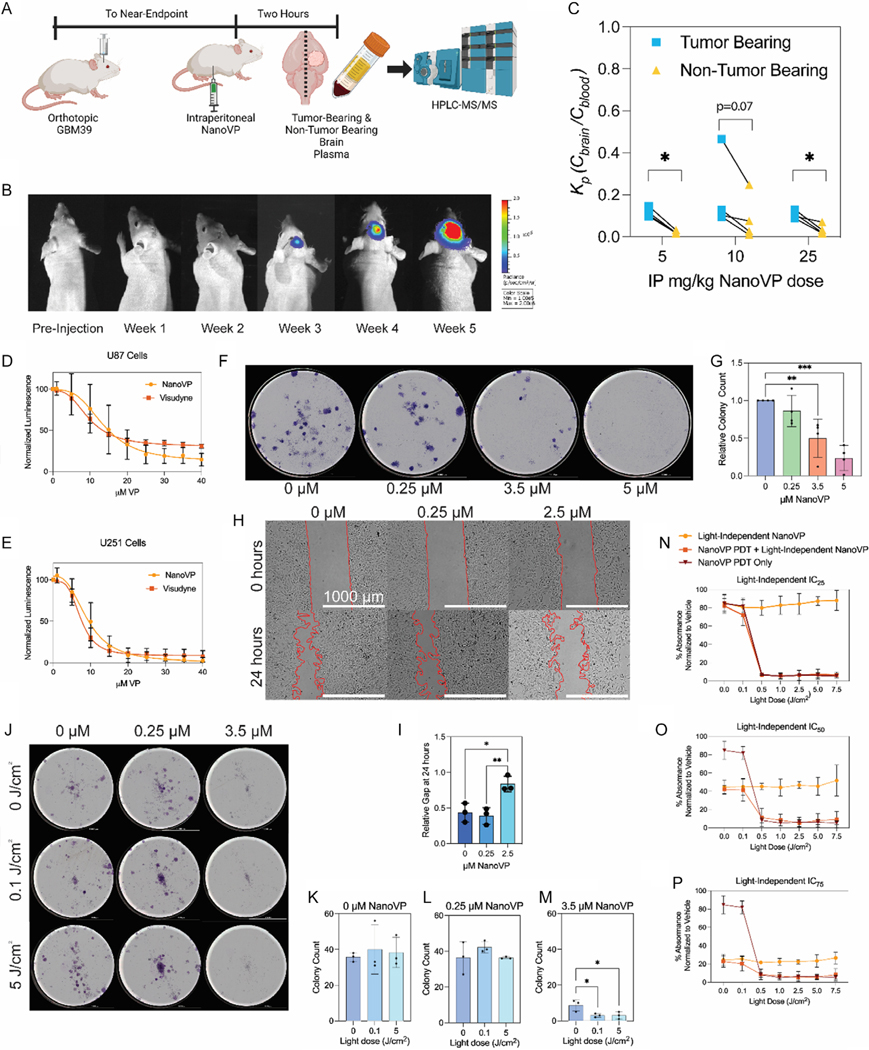
Efficacy of NanoVP in the context of glioblastoma. A) To evaluate the bioavailability of NanoVP, mice bearing near-endpoint orthotopic GBM39 tumors received 5, 10, or 25 mg kg^−1^ IP injections of NanoVP. Two hours later, mice were euthanized, and brains and plasma were collected for HPLC-MS/MS analysis. B) IVIS images demonstrate relative size and sidedness of the GBM39 tumors over time. C) The *K*_p_ value, or the concentration of VP in each hemisphere of the brain divided by the plasma concentration, reveals a higher accumulation of VP in the tumor-bearing hemisphere compared to the nontumor-bearing hemisphere. To evaluate light-independent efficacy of VP, CellTiter Glo was used to estimate viability in D) U87 and E) U251 cells was assessed after 72 h of incubation with either NanoVP or a Visudyne-like formulation of liposomal VP. NanoVP and Visudyne-like VP performed similarly. F) Clonogenic assays (160 cells well^−1^) and G) quantification revealed a dose-dependent decrease in clonogenic capacity in U251 cells after 6 days of incubation. H) A gap closure assay, where red lines indicate gap boundaries and the white bar indicates scale (1000 μm), and I) quantification revealed decreased movement of U251 cells after 24 h of incubation with drug. To combine NanoVP-PDT with light-independent effects of NanoVP, J) a clonogenic assay on U251 cells was performed where cells received NanoVP PDT (0.25 μM NanoVP for 90 min, washed twice with PBS before light irradiation (690 nm, 10 mW cm^−2^, 0–5J cm^−2^)). The next day, 160 viable cells/well were seeded and allowed to adhere for 24 h. Cells were then incubated with NanoVP in the dark (0–3.5 μM) for 6 days. When quantified, no combinatorial effect was observed with K) 0 μM or L) 0.25 μM light-independent NanoVP, but a significant decrease in colony count was observed with M) 3.5 μM light-independent NanoVP in groups that also received PDT. When U87 cells received PDT (0.25 μM NanoVP for 90 min, washed twice with PBS before light irradiation (690 nm, 10 mW cm^−2^, 0–7.5 J cm^−2^)) then light-independent NanoVP at their N) IC_25_, O) IC_50_, or P) IC_75_ for 72 h before MTT assay, no combinatorial effect was observed, and cell killing was dictated by either the light-independent NanoVP or the NanoVP-PDT. For (C), significance was determined by *t*-test, *n* = 4. Condition 4 NanoVP (Table 1) was used for (A,B,C, D,E,N,O,P). Condition 2 NanoVP (Table 1) was used for (F–M). For (G,I,K,L,M), significance was determined via one-way ANOVA with Tukey’s multiple comparisons against the control. *p < 0.05, **p < 0.01, and ***p < 0.001. Graphic (A) made with BioRender.

Verteporfin has recently returned to clinical interest for its potential to manage cancer, particularly glioblastoma, in a light-independent manner.^[60]^ As such, we evaluated both the bioavailability and activity of NanoVP in the context of GBM. First, mice with near-endpoint orthotopic GBM39 tumors received 5, 10, or 25 mg kg^−1^ via IP injection (Figure 9A). Two hours postinjection, the mice were euthanized before plasma and both brain hemispheres were collected. VP levels were quantified via high-performance liquid chromatography-tandem mass spectrometry (HPLC-MS/MS). At this point, tumors were quite large and right-sided (Figure 9B). After quantification, the levels of VP in the plasma and hemispheres were summarized as *K*_p_, which is the ratio between brain concentrations and plasma concentrations of the drug.^[61]^ The ratios were similar between all injected doses, with a higher ratio in the tumor-bearing hemisphere (Figure 9C). Raw serum and brain values are provided in Table S2, Supporting Information.

Next, we evaluated the potential of NanoVP to inhibit the growth and migration of GBM cells in vitro in the absence of light. First, we formulated a liposomal formulation of VP like Visudyne (Figure S1A,B, Supporting Information)^[46]^ to use as a control. DLS analysis revealed that this formulation had a wide range of diameters (1010 ± 1210 nm; Figure S1C,D, Supporting Information), a Pdi > 0.2, and a negative surface charge (Figure S1E, Supporting Information). First, we treated cells with Visudyne-like VP and NanoVP for 72 h and then assessed viability via CellTiter Glo (Figure 9D,E). In U87s and U251s, the Visudyne-like formulation and NanoVP performed similarly. For U87 cells, the IC_50_s were 13 ± 3.5 μM for NanoVP and 9.8 ± 0.62 for Visudyne-like VP (*p* = 0.16 by two-tailed Student’s *t*-test). For U251 cells, the IC_50_s were 9.1 ± 1.87 for NanoVP and 7.4 ± 0.80 for Visudyne-like VP (*p* = 0.22 by two-tailed Student’s *t*-test). Next, we evaluated the ability of NanoVP to inhibit the clonogenic capacity of U251 cells by plating U251 cells at a low density and treating them with NanoVP for 6 days (Figure 9F,G). Normalized colony count indicated a decrease in clonogenic capacity at concentrations higher than 0.25 μM, which is a concentration that is efficacious for PDT.^[40]^ We also evaluated the ability of NanoVP to inhibit gap closure in U251 cells, where cells were seeded on either side of a silicone insert and then allowed to migrate for 24 h in the presence of NanoVP (Figure 9H,I). We observed a decrease in gap closure at concentrations above the dose that is efficacious for PDT, which is 0.25 μM.

Because VP has been used in the clinic for over 20 years as a PDT agent,^[62]^ we evaluated the impact of combined PDT and light-independent application of NanoVP. First, we performed PDT on cells and then plated only viable cells for clonogenic evaluation. After adhering for 24 h, cells were treated with 0.25 or 3.5 μM NanoVP in the dark for 6 days. In U251 cell, we found that there was a significant decrease in clonogenic capacity after PDT at levels greater than the PDT-dose light-independent control (Figure 9J–M). Even with low PDT doses of 0.1 J cm^−2^, there was decreased clonogenic capacity in the group treated with 3.5 μM NanoVP in the absence of light. We also evaluated the combination of PDT and dark effects of VP in U87 cells. In this assay, cells were plated, PDT was performed, and then cells were treated with the light-independent IC_25_, IC_50_, or IC_75_ for NanoVP on U87 cells.^[40]^ Via MTT assay, we found no combinatorial effect between PDT and the light-independent application of NanoVP in U87 cells (Figure 9N–P), with cell viability being driven, apparently, by either PDT or light-independent effects of VP.

## 3. Discussion

Verteporfin has been clinically useful in PDT for wet age-related macular degeneration (AMD) since its FDA approval in 2002. Our main motivation for developing this formulation was the new application of VP as a chemotherapy agent, which requires higher dosing over a longer period of time than what is traditionally required for PDT. PDT for AMD involves a single infusion of Visudyne at 6 mg m^−2^ followed by light activation.^[27]^ In current clinical trials of VP for GBM management in the absence of light, Visudyne is infused over 83 min weekly for ≈11 weeks (NCT04590664). This repeated dosing underscores the need to re-evaluate the delivery of VP with lipid carriers. While nanoparticle-based delivery does permit targeted delivery and reduces side effects for several drugs, it comes with undesirable tradeoffs. Carriers can induce anticarrier immunity^[7]^ and even commonly used excipients can induce hypotension,^[63]^ hERG inhibition,^[64]^ hepatotoxicity,^[65]^ and nonspecific BBB permeabilization.^[66]^ Carriers can reduce toxicity, such as the case of Abraxane, but it ultimately performs the same as solvent-based paclitaxel in many cases.^[67]^ Recent studies have found that the VP in Visudyne quickly redistributes to plasma molecules^[50]^ and our results indicate that NanoVP may perform similarly.^[40]^ While these kinetics require further investigation, the lipids in Visudyne may ultimately not be needed to achieve similar results. Ultimately, many hydrophobic drugs fail to make it to the clinic due to solubility issues, and the hydrophilic drugs that do make it to the clinic rarely require carrier systems. Carrier-free solutions represent the majority of clinically used drugs, and nanoparticle-encapsulated drugs like Visudyne are the exception. VP’s aggregation in aqueous solution has, to date, prohibited its delivery as a free drug. Large, uncontrolled aggregation of drug is undesirable for clinical delivery as surface area can vary significantly, resulting in aberrant drug release and unpredictable bioavailability and pharmacokinetics. In contrast, the release of drug from nanosuspension formulations is more predictable and generally faster due to increased surface area, permitting release predicted by the Noyes–Whitney equation.^[68]^ Here, we have tuned the concentration, diameter, and zeta potential of nanoaggregates suitable for infusion.

Broadly, nanosuspensions are formulated either by “top–down” techniques, where large particles are broken down into smaller particles, or “bottom–up” techniques, where the nanosuspension is formulated from a molecular dispersion.^[69]^ Bottom-up processes are generally thought to be simpler, require less energy, and require simpler tool than top–down processes and can result in more monodisperse and smaller populations of particles.^[70]^ While the potential toxicity of stabilizers needed to maintain a nanosuspension is a concern,^[71]^ it is feasible to create stable nanosuspensions without the use of stabilizers, permitting carrier-free delivery of hydrophobic drugs, as we have done with NanoVP.

We observed that DMSO produced monodisperse nanoaggregates without the need for filtration. Acetone produced a slightly more polydisperse population and required filtration to reduce the range of particle sizes. We observed uncontrolled aggregation with clear precipitation of large aggregates when we attempted to make particles with ACN, EtOH, or MeOH. While purification via filtration did result, in most cases, in clean peaks, the material loss associated with these solvents was too great to warrant further investigation. Others have reported that materials with low water solubility have higher diffusivity within the more polar solvent phase (e.g., VP in DMSO), while higher viscosity of the solvent may limit mixing with aqueous solution.^[72]^ From this, rapid movement of VP within DMSO balanced with slower mixing of DMSO with water may permit a favorable balance of nucleation and growth, resulting in consistently sized particles. Other solvents may have failed to provide a balanced nucleation and growth rate. For example, EtOH and MeOH were near maximal saturation when mixed with water, so there was likely no controlled rate of nucleation or growth, resulting in the formation of primarily large aggregates.

Our initial formulation of NanoVP resulted in a roughly spherical amorphous nanoaggregate of verteporfin using a robust and reproducible synthesis scheme. From this, we identified factors important to the formulation of NanoVP: three that modulate the diameter of the nanoformulation, three that altered the Pdi, two that altered peakedness of particles, and two that were inconsequential to NanoVP characteristics. In solvent–antisolvent precipitation, particle diameter is a result of a balance between nucleation and growth. Nucleation is the process by which initial aggregates form spontaneously or around contaminants in solution. Growth is the process by which monomers join existing aggregate sites.^[57,73]^ This process is thought to be dependent on the local concentration of supersaturated particles, permitting nucleation, and the flux of monomers surrounding nucleation sites. Factors that reduced the diameter of particles (e.g., increased stirring speed, increased droplet height, decreased VP concentration in DMSO) likely increased the flux of monomers around nucleation sites, resulting in smaller diameter particles. Alternatively, the mixing of miscible solvent and antisolvent can be considered in terms of mesomixing (bulk mixing of the two fluids) and micromixing (diffusion of one fluid in another). Increasing stirring speed likely increased the rate of micromixing, as stirring increased the rate of diffusion of VP in eddies of DMSO throughout the bulk of the water.^[74]^ As such, nucleation sites were likely dispersed throughout the solution, resulting in more opportunities for *de novo* nucleation site formation rather than growth with high stirring speeds. Droplet height was also found to be inversely proportional to particle diameter. In this instance, the increased speed of droplets of VP in DMSO interacting with water likely led to a greater dispersion of VP in DMSO eddies in water, resulting, once again, in a process favoring nucleation over particle growth. Consistent with this interpretation, increased concentration of VP in DMSO resulted in larger particles, likely because equal mixing provided equal opportunity for nucleation initiation but increased local concentrations of VP in antisolvent without greater dispersion favored growth over additional nucleation. In this case, an increased concentration of VP in DMSO likely led to incomplete mixing and ultimately increased nanoparticle size.^[70]^

We observed decreased polydispersity with increased depth of water, and broad peaks on DLS with shallow water and both low and high volumes of water. This may be due to the initiation of nucleation processes. It is thought that nucleation can be either homogenous, where particles spontaneously aggregate with their own species, or heterogeneous, where contaminants, even in the cleanest of systems, initiate nucleation.^[56]^ Practically, nucleation of NanoVP is likely due to heterogenous initiation. In the cases of shallow water and low water volume, the walls of the container may also serve as nucleation sites, resulting in less controlled nucleation and aggregation. Conversely, a large volume of antisolvent may disperse monomers too broadly, again resulting in aberrant nucleation and growth.

By combining parameters, we were able to tune NanoVP diameter from 49.0 ± 4.4 to 195 ± 7.1 nm, representing a ≈64-fold increase in volume, assuming a spherical particle. Interestingly, increasing the value of a single parameter (stirring speed) reduced the diameter of particles from 195 ± 7.1 to 63.8 ± 5.0 nm. This is likely due to increased dispersion of monomers, resulting in higher nucleation rates and decreased growth rates, particularly because the largest particles were formulated in still water (0 RPM). The largest particles had a broader distribution on TEM, which was incongruous with a lower Pdi value from DLS. Under all formulation parameters, particles remained amorphous, likely because the mechanisms of nucleation and growth did not significantly change with the parameters studied.

When increasing the diameter of particles, we observed that the zeta potential decreased. Upon compiling all data regarding diameter and zeta potential, we found an indirect relationship between diameter and charge. Zeta potential is an inferred charge on the surface of a particle based on the concentration of ions present on the border between the nanoparticle-associated Stern layer and the bulk phase of the liquid. It is possible that the carboxylic acid groups on VP faces the aqueous solution in NanoVP, explaining the more negative charge with increased diameter. This is consistent with the finding that NanoVP loses stability in solutions below its pKa, potentially indicating that, when the carboxylic acid is proton bound, NanoVP loses its negative surface charge and dissociates. The lack of stability in pHs below 4.5 partially explains the lack of stability in certain media.

Under the formulation parameters explored here, Condition 4 best meets our design requirements. It had a high overall VP concentration but retained a small and monodisperse particle size. While previous work primarily used Condition 2 NanoVP,^[40]^ we envision future work, especially in vivo studies, using this higher-concentration, smaller-diameter formulation.

It is worth noting that parameters were explored with handmade samples, and within these samples, we observed from 8% (Condition 3) to 18% (Condition 1) variability in diameter and between 11% (Conditions 3 and 4) and 114% (Condition 1) variability in concentration with samples made by two welltrained experimenters. In order to reduce this variability, and foreseeing a need for larger volumes of NanoVP samples, we evaluated automated production of NanoVP. Our aim was to increase consistency between samples by automating the pump system. First, we established that pump rate did not impact size, peakedness, Pdi, or zeta potential for produced NanoVP. We hypothesize that this is because, under all conditions (manual and pump produced), droplets of VP in DMSO accumulated on the tip of the needle and pipette, so the introduction to antisolvent was under approximately the same parameters, influenced primarily by acceleration of the droplet by gravity from a constant height above the surface of the water. We compared the diameter, Pdi, and zeta potential of four automated pump samples at each pump rate and four random handmade samples. We found no significant changes in diameter, Pdi, or zeta potential, indicating that the pump system is as consistent as our handmade protocol, but not more so, for Condition 2 NanoVP nanoparticles.

Finally, we utilized an in vivo flank-passaged patient-derived orthotopic xenograft of GBM, GBM39,^[75]^ to evaluate bioavailability of VP in the brain after large-dose IP administration of NanoVP. We previously reported that NanoVP, injected at 0.5 mg kg^−1^, has a pharmacokinetic profile similar to Visudyne and accumulates nonspecifically in heavily vascular-ized tissues as well as the tumor and the brain.^[40]^ VP doses in the 5–100 mg kg^−1^ range in mice have been used to observe anticancer light-independent effects of the drug.^[32,33]^ We waited until near-endpoint to dose mice with IP NanoVP in an attempt to recapitulate a potentially leaky blood–brain barrier (BBB) in the tumor-bearing hemisphere, but to also be able to quantify VP penetration in the nontumor bearing hemisphere, which presumably should have an intact BBB.^[76]^ We reported *K*_p_, a ratio of total brain concentration to total plasma concentration. This measure provides a high-level overview of the impact of drug protein binding in the blood, drug binding to brain cells and proteins, and transport (both active and passive) across the BBB.^[61]^ The difference in *K*_p_ between the tumor-bearing and nontumor-bearing hemisphere indicates a difference in one of these processes, most likely in either drug binding to brain cells and proteins or transport across the BBB as binding to plasma proteins should be consistent between hemispheres within the same mouse.

Drugs that cross the BBB generally have a molecular weight of less than 500 Da, are lipophilic but not so much so that partitioning into the aqueous brain stroma is prohibited, and are not actively effluxed from the brain stroma to the blood the brain.^[77]^ VP has a molecular weight of 718.8 Da, is lipophilic, and is a substrate of ATP-Binding Cassette (ABC) transporters at the BBB,^[78]^ meaning that it is unlikely that the drug crosses an intact BBB. Generally, a drug with a *K*_p_ < 0.3 is considered to be poorly bioavailable in the brain.^[79]^ In this study, we found *K*_p_ values in both the tumor-bearing and nontumor-bearing hemispheres below 0.3 in all mice but one. Consistent with theory, it appears that VP poorly enters the CNS. Still, enough enters the brain to see efficacy in mice^[32]^ and measurable levels in humans.^[33]^ In this study, IP injection was selected due to limitations on bolus injectable intravenous volume. The similarity in concentrations between doses may be attributable to the saturation of absorption and transportation from the peritoneal cavity to the blood and ultimately to the brain.

Still, we observed an increase in *K*_p_ in the tumor-bearing hemisphere compared to the nontumor-bearing hemisphere in the 5 mg kg^−1^ (5.7-fold increase in *K*_p_, *p* = 0.001), 10 mg kg^−1^ (4.6-fold increase, *p* = 0.07), and 25 mg kg^−1^ (4.0-fold increase, *p* = 0.02) treatment groups. This may be attributable to the tumor’s impact on the vasculature, or the interaction between VP and tumor cells. The large tumor used in this study may exhibit enhanced permeability and retention, where irregular neovascularization, elevated inflammatory cytokines, and poor lymphatic drainage may lead to enhanced accumulation of VP at the tumor site.^[17]^ In the context of GBM, this blood–tumor barrier (BTB) has unique properties distinct from the BBB^[80]^ and may explain the observed increase in VP in the tumor-bearing hemisphere. There is also a possibility that the increased accumulation in the tumor-bearing hemisphere is attributable to improved accumulation within tumor cells. Previous work has shown that VP in the blood interacts with various blood biomolecules, which may have preferential uptake by tumor cells.^[81]^ While this uptake may not translate directly, factoring in the additional absorption from the peritoneal cavity to the blood, considering the partitioning of VP in the blood as a potential driver of accumulation within the tumor-bearing hemisphere may be worthwhile. Previously, we reported that NanoVP dissociates in the presence of serum proteins in a time-dependent and concentration-dependent manner.^[40]^ There are a few hypotheses for how NanoVP might cross the BBB. NanoVP may dissociate completely in the blood and passively cross the BBB as monomeric VP. NanoVP may also only partially dissociate, creating a mix of monomers and smaller NanoVP particles that passively cross the BBB. Based on the data presented here, it seems that only the disrupted BBB permits VP accumulation within the brain. Further work is needed to understand both the kinetics of NanoVP in the blood and what form of NanoVP reaches the brain parenchyma.

Finally, we evaluated the ability of NanoVP to impact cell proliferation, clonogenic capacity, and motility and examined the interaction between sequential PDT and light-independent activity of the NanoVP. We found that NanoVP performed similarly in killing cells to a Visudyne-like formulation of VP. As the two formulations performed similarly, and considering shortages of Visudyne,^[51]^ we proceeded to evaluate the capacity of NanoVP alone as others have outlined the capacity of Visudyne to perform in a light-independent manner.^[33]^ We observed a reduction of clonogenic capacity and motility in U251 glioblastoma cells at sub-IC_50_ concentrations of the drug. While further work is needed to confirm that these changes to cellular behavior are YAP dependent, these robust and promising functional findings are consistent with expanding literature that indicates light-independent VP may be a good therapeutic option. An important control in these studies was the inclusion of light-independent VP at a concentration of 0.25 μM, a low concentration of the drug that is efficacious for PDT. We observed no light-independent treatment effect in the 0.25 μM group, indicating that current PDT practices with VP may not induce any light-independent effects, and a separate dosing regimen may be required. We then evaluated the combination of PDT with NanoVP treatment in a light-independent manner. In a clonogenic assay, where only viable U251 cells were replated post-PDT, we observed a significant decrease in clonogenic capacity in all groups that received PDT treatment. We did not observe a combinatorial effect in U87 cells treated with a similar combination as measured via MTT assay, potentially because the mitochondrial activity of the PDT-treated cells^[82]^ may have recovered by the time the assay was performed.^[83]^

Others have commented on the potential to combine VP-PDT with light-independent effects of the drug^[84]^ and have characterized the PDT response of GBM cells to complement studies on light-independent effects.^[85]^ Some work has been done to evaluate the combinatorial effect of these two mechanisms,^[86]^ although it is missing the high daily dosing present in other studies evaluating the light-independent effects of VP.^[32,33]^ To our knowledge, this study presents the first combination of VP-PDT with light-independent VP with appropriate dosing to elicit both effects. While the clonogenic data is promising, this combinatorial study was limited by the order in which VP-PDT and light-independent VP were applied and by the system and assays used. The concentrations of VP for light-independent effects were an order of magnitude higher than those for PDT. We only performed PDT before providing enough VP for light-independent effects to avoid over-sensitizing cells to light. As such, we were unable to assess the potential of light-independent application of VP to sensitize cells to PDT. Further combinatorial studies, including expanded duration of light-independent VP exposure and order of VP-PDT and light-independent VP exposure (e.g., light-independent effects then PDT, or light-independent then PDT then light-independent again), are worth exploring. Results in this study also indicated the VP poorly penetrates the brain. It is worth considering that PDT-mediated BBB permeabilization^[40]^ may enhance the delivery of VP to the brain to enable enhanced light-independent effects. As such, biodistribution studies after BBB permeabilization as well as transwell assay models of permeabilization^[87]^ may also inform the combination of PDT and light-independent applications of VP.

Here, we identified key parameters in the synthesis and stability of NanoVP. We managed a twofold increase in diameter and sixfold increase in concentration. We found that our largest particles had a significantly more negative charge than our initial formulation. The utility of the varied diameter in biological contexts warrants further evaluation. The −30 mV threshold is typically thought to confer greater stability to particles in solution due to increased interparticle repulsive forces, so future work may compare the biological impacts of large, very negative particles compared to smaller, less negatively charged particles. Future work will compare the stability and bioactivity of large, very negative particles and smaller, less negatively charged particles, including their light-dependent and light-independent activity, pharmacokinetics, and biodistribution. Because the dissociation and uptake kinetics of these differently sized and differently charged nanoparticles may vary, future work will also evaluate in vitro differences in the behavior of these different particles.

A further limitation of the current study is that all samples, barring pump-made samples, were produced by hand. Future work will expand on the scale-up of the pump-made system, as well as other considerations for the clinical application of NanoVP, including endotoxin testing. Through brain accumulation studies, we found that VP may be minimally bioavailable in the brain. Future work may evaluate the brain biodistribution more carefully, including establishing the *K*_p,uu,brain_, which establishes the amount of unbound, bioactive drug in the brain compartment.^[79]^ In addition, the specific partitioning of NanoVP in the blood, especially in contrast to liposomal formulations of the drug,^[48,49,88]^ warrants further and careful investigation beyond the existing evidence that NanoVP dissociates and becomes bioavailable and photoactive in the presence of biomolecules.^[40]^ Finally, our results surrounding the light-independent application of NanoVP are consistent with others’ reports that VP is cytotoxic and inhibits cell motility.^[32,60]^ The combination of PDT with NanoVP and the light-independent effects of NanoVP shows some slight combinatorial effect; however, combining PDT in vivo for BBB or BTB permeabilization^[40]^ to enhance VP delivery to the brain and subsequently enhance light-independent effects may hold promise. Additionally, the evaluation of cell lines dependent on YAP-mediated signaling, particularly glioma stem-like cells,^[89]^ may show greater efficacy than the studies shown here. In all, NanoVP represents an attractive option for the clinical delivery of a hydrophobic drug, especially considering the current interest in repurposing VP for light-independent applications.

## 4. Experimental Section

### Chemicals:

VP (also known as BPD-MA) was acquired from US Pharmacopeia (Rockville, MD, USA). UltraPure distilled water was acquired from Invitrogen (Life Technologies, Grand Island, NY, USA). Dimethyl sulfoxide (DMSO) was acquired from Fisher Chemical (Waltham, MA, USA). Acetone was acquired from Fisher Chemical (Waltham, MA, USA). EtOH was acquired from Pharmco (Brookfield, CT, USA). HPLC-grade MeOH was acquired from Alfa Aesar (Ward Hill, MA). Calcbiochem OmniPur 10X PBS concentrate was acquired from Millipore (Burlington, MA, USA) and diluted to 1X in deionized water. Instant Ocean was acquired from Spectrum Brands (Blacksburg, VA, USA) and dissolved in water at a working concentration in deionized water. ACN, dextrose, sodium chloride solution (5 M, diluted to 0.9 w/v% in deionized water), HPLC-grade water, crystal violet, chloroform, and 10% formalin were acquired from Sigma–Aldrich (St. Louis, MO, USA).

### NanoVP Formulation:

Verteporfin dissolved in DMSO was added dropwise with a 10 μL pipette to UltraPure water stirred with a 10 × 3 mm stir bar (Fisher Scientific, Waltham, MA, USA) on a Cimarec + Stirring Hotplate (7.25” × 7.25” or 10.25” × 10.25”) (Thermo Scientific, Waltham, MA, USA) over a 5 min period. After all VP in DMSO was added, the nanosuspension was allowed to mix until homogenous (≈3 min), then transferred to Spectra/Por 300 kD Cellulose Ester Float-A-Lyzer G2 dialysis device or Spectra/Por 300 kD Biotech Cellulose Ester Tubing (Repligen, Waltham, MA, USA) and dialyzed against PBS for 24 h at 4 °C. VP concentration was determined based on UV-Vis absorbance in DMSO (Synergy Neo2, BioTek Instruments, Winooski, Vermont, USA) using the established molar extinction coefficient (VP: ε = 80 500 M^−1^ cm^−1^ at 435 nm, ε = 34 895 M^−1^ cm^−1^ at 687 nm).

### Dynamic Light Scattering:

Hydrodynamic diameter, polydispersity index (Pdi), and zeta potential were measured using a particle sizer and zeta potential analyzer (NanoBrook Omni, Brookhaven Instruments, Holtsville, NY, USA). For hydrodynamic diameter and Pdi, 20 μL NanoVP was mixed with 2000 μL UltraPure water. In the case of very low concentrations of sample (e.g., 1% solvent-to-antisolvent ratio; 1 mM VP in DMSO; ACN, EtOH, and MeOH samples after filtration), the full volume used was sample. For zeta potential, 20 μL sample was mixed with 1600 μL 10 mM sodium chloride.

### Transmission Electron Microscopy:

NanoVP samples were either made fresh and dialyzed against deionized water before transmission electron microscopy (TEM) or dialyzed from PBS against deionized water to remove salts from the sample. The size, morphology, and microstructure of NanoVP were studied using a TEM (JEOL JEM-2100 LaB6, 200 kV; Peabody, MA, USA). For conventional TEM, NanoVP (10 μL) was pipetted onto a Lacey carbon grid (Ted Pella, Redding, CA, USA) and air dried overnight before the examination. Images were taken at high magnifications (10 000–1 00 000x). Due to small particle sizes and to enhance diffraction intensity, selected area electron diffraction was used to collect electron diffraction patterns.

### Automated Pump System:

An NE-1600 Syringe Pump (New Era Pump Systems, Inc., Farmingdale, NY, USA) was affixed to a 3 mL BD Plastipak Luer-Lok syringe (Becton, Dickinson and Company, Franklin Lakes, NJ, USA). The syringe was affixed to a female luer lock with 1/8” inner diameter (I.D.) polypropylene barb adapter (Avantor Masterflex, VWR LLC, Radnor, PA, USA). The barb adapter was connected to ≈30 cm of 1/8” I.D. polypropylene tubing (United States Plastic Corp, Lima, OH, USA), which was connected to a 1/8” I.D. to male polypropylene luer lock barb adapter (RSN Lab Store). The male luer lock was attached to a 20-gauge blunt tip polypropylene female luer lock hub needle (Jensen Global, Santa Barbara, CA, USA).

### Animal Studies:

Animal protocols (R-MAR-22–16 and R-MAY-23–27) were approved by the University of Maryland, College Park Institutional Animal Care and Use Committee. GBM39 was passaged in the flank of J:Nu female mice (4–5 weeks old, #007850, The Jackson Laboratory, Bar Harbor, ME, USA). Subcutaneous GBM39 tumors were dissociated using a gentleMACS tumor dissociation kit (Miltenyi Biotec, Auburn, CA, USA). 5 × 10^5^ cells in 5 μL PBS were stereotactically injected using a Hamilton syringe (Hamilton Company, Reno, NV, USA) at 2 mm right lateral to bregma and 3 mm deep in female J:Nu mice (4–5 weeks old). When mice were near endpoint based on weight and a physical examination score sheet, mice received 0, 5, 10, or 25 mg kg^−1^ NanoVP (Condition 4, Table 1) via IP injection. Two hours later, mice were euthanized via CO_2_ asphyxiation and cervical dislocation. Blood was collected via cardiac puncture and placed in a heparin tube (Sarstedt, Inc., Numbrecht, GER) and then centrifuged to isolate plasma according to manufacturer recommendations before storage at −80 °C. The left (nontumor-bearing) and right (tumor-bearing) brain hemispheres were stored at −80 °C.

### Biodistribution and Mass Spectrometry:

Brain sections were homogenized with a BeadBug 6 (Benchmark Scientific, Sayreville, NJ, USA) in 2.0 mL tubes prefilled with 3.0 mm zirconium homogenizer beads (Stellar Scientific, Baltimore, MD, USA). Brain sections were mixed 1:3 w/w with HPLC-grade water for homogenization. Both homogenized brain and plasma were mixed 1:8:1 sample: ACN: MeOH v/v. Mixed samples were vortexed and then stored at −80 °C for at least 30 min before centrifugation at 21.1 g for 20 min. Supernatants were transferred to centrifugal polytetrafluoroethylene filters (MilliporeSigma, Burlington, MA, USA) and centrifuged according to manufacturer protocols. Flow-through was transferred to HPLC tubes (Waters Corporation, Milford, MA). Waters H-Class UPLC/Xevo TQD mass spectrometer was used for quantification of VP. Samples were analyzed using ACQUITY UPLC BEH reversed phase C18 column (130 Å, 1.7 μm, 2.1 × 50 mm; Waters Corporation, Milford, MA). At a flow rate of 0.4 mL min^−1^, liquid chromatography was performed where A was water with 0.1% formic acid and B was ACN with 0.1% formic acid: 20% A from 0 to 1 min; 20% A to 0% A from 1 to 3.2 min; 0% A to 20% A from 3.2 to 3.3 min; 20% A from 3.3 to 3.5 min. VP eluted as ion two peaks at ≈3.02 and ≈3.11 min. Daughter ions detected were 645.45, 513.37, and 499.93 m z^−1^.

### Cell Viability Studies:

The human glioblastoma cell lines U87 and U251 were obtained from ATCC and cultured per the vendor’s instructions. Both cell lines were cultured in Eagle’s Minimum Essential Medium (Cellgro, Lincoln, NE, USA) with 10% v/v fetal bovine serum (Gibco), 100 U mL^−1^ penicillin, and 100 μL mL^−1^ streptomycin. Cells were maintained in 5% CO_2_ at 37 °C and were regularly monitored for mycoplasma according to manufacturer protocols (MycoAlert, Lonza, Basel, CH).

For PDT studies, cells were plated in black-walled, clear bottom plates or 35 mm Petri dishes (1–3.3 × 10^4^ cells cm^−2^) and allowed to adhere for 24 h before incubation with NanoVP (0.25 μM, Condition 4, Table 1) for 90 min. Cells were washed with PBS twice immediately before light irradiation (0–7.5 J cm^−2^, 10 mW cm^−2^, bottom illumination; ML6600, Modulight Corporation, Tampere, FIN).

For light-independent VP viability studies, cells were incubated with NanoVP (Condition 4, Table 1) or Visudyne-like VP for 72 h before viability was measured via CellTiter Glo (Promega, Madison, WI, USA) or 3-(4,5-dimethylthiazol-2-yl)-2.5-diphenyltetrazolium bromide (MTT) assay (Sigma–Aldrich, St. Louis, MO, USA) according to manufacturer’s protocols. Luminescence (CellTiter Glo) or absorbance at 580 nm (MTT) were measured via plate reader (Synergy Neo2, BioTek Instruments, Winooski, Vermont, USA).

### Clonogenic Assays:

U251 cells were seeded at a density of 45 cells cm^−2^ (160 cells well^−1^) in a 12-well dish. Cells were allowed to adhere for 24 h. Cells were incubated with NanoVP (0–5 μM, Condition 2, Table 1) for 6 days. Cells were fixed with 10% formalin for 20 min, then stained with 0.1% crystal violet for 20 min, and rinsed three times with PBS. Wells were imaged on a LionHeart plate imager (BioTek Instruments, Winooski, Vermont, USA) and colonies were manually counted in ImageJ. For clonogenic assays after PDT, viable cells were counted using propidium iodide staining, and 45 cells cm^−12^ of viable cells were plated in 12-well dishes for clonogenic development.

### Gap Closure Assay:

U251 cells were seeded in two-well silicone culture inserts (ibidi USA, Inc., Fitchburg, Wisconsin, USA) at a density of 50 000 cells cm^−2^ (10 000 cells per well) and allowed to adhere for 24 h. Before removal of the insert, cells were treated with mitomycin C (AdooQ Bioscience, Irvine, CA, USA) at a concentration of 10 μg mL^−1^ for 1 h. Inserts were removed and cells were treated with 0–2.5 μM NanoVP (Condition 2, Table 1). Gaps were imaged on a LionHeart imager at 0 and 24 h postinsert removal. Gap area was quantified using ImageJ.

### Visudyne-like Verteporffn Formulation:

Verteporfin, 1,2-dimyristoylsn-glycero-3-phosphocholine (DMPC, Avanti Polar Lipids, Alabaster, AL, USA), L-α-phosphatidylglycerol (Egg PG, Avanti Polar Lipids, Alabaster, AL, USA), [16-[(7-nitro-2–1,3-benzoxadiazol-4-yl)amino] palmitic acid (NBD Palmitic Acid, Avanti Polar Lipids, Alabaster, AL, USA), and butylhydroxytoluene (MilliporeSigma, Burlington, MA, USA) were mixed in a 1:3:5:0.1:0.1 molar ratio in chloroform. The mixture was flash-frozen via submersion in liquid nitrogen. Chloroform was removed from the sample via lyophilization on a FreeZone 6 L Console Freeze Dryer (Labconoco Corp., Kansas City, MO, USA). Samples were stored at −20 °C in the dark until use, at which point they were vortexed with sterile PBS containing calcium and magnesium.

### Statistics:

Statistical tests were carried out using GraphPad Prism (GraphPad Software, San Diego, CA, USA) or Microsoft Excel (Microsoft, Redmond, WA, USA). For nanoparticle parameter descriptions, one-way ANOVA statistical tests with Tukey posthoc tests were performed. For DLS diameter curves, data are presented as mean ± standard deviation. For TEM image quantification, particle diameter was measured using ImageJ, and particle frequency (defined as the frequency of values within a specific histogram bin) was plotted, with a nonlinear fit curve overlayed. For Pdi and zeta potential, the central bar indicates the mean, with boxes extending to minimum and maximum values. For diameter cartoons, mean is shown as a thin-lined black circle, with standard deviation represented as green or gray concentric circles. For pH response, raw values are shown. For concentration, data is shown as mean ± standard deviation. For relationships between hydrodynamic diameter, Pdi, and zeta potential, best-fit lines were produced by simple linear regression. Dotted lines indicate the confidence interval. For brain biodistribution, *K*_p_ is shown, which is the concentration of VP in the brain divided by the concentration of VP in the plasma. Two-tailed Student’s *t*-tests were used to compare the tumor-bearing and nontumor-bearing values within each treatment group. Each mouse’s tumor-bearing and nontumor-bearing values are connected by a black line. For other biological assays, data is presented as mean ±standard deviation, and significance was determined by one-way ANOVA with Tukey’s multiple comparisons against the control group, or a two-tailed Student’s *t*-test. All data presented is representative of at least three replicates.

## Supplementary Material

Suppl

Supporting Information

Supporting Information is available from the Wiley Online Library or from the author.

## Figures and Tables

**Table 1.** Specific formulation parameters for NanoVP.

Parameter	Range tested	Condition 1	Condition 2 (initial)	Condition 3	Condition 4 (ideal)	Design criteria	Visudyne^[45,46]^

Droplet volume [μL]	10–100	10	10	10	10	–	–
Droplet height [cm]	1–15	15	5	1	15	–	–
Stirring speed [RPM]	0–1500	1500	400	0	1500	–	–
Antisolvent temperature [°C]	4–80	20	20	20	20	–	–
VP in solvent [mM]	1–15	1	7	15	15	–	–
Solvent-to-antisolvent ratio [%]	1–6	1	2	6	6	–	–
Volume of water [mL]	1–25	5	5	5	5	–	–
Depth of water [mm]	2–8	4	4	4	8	–	–
Diameter [nm]	–	49.0 ± 4.4	104 ± 6.0	195 ± 7.1	63.8 ± 5.0	<64	524.8 ± 547.5; 733.8 ± 488.9
Concentration [μM]	–	9.1 ± 5.2	136 ± 10	838 ± 45	799 ± 44	>500	2780 (Theoretical)
Pdi	–	0.21 ± 0.02	0.12 ± 0.01	0.12 ± 0.02	0.14 ± 0.01	<0.20	0.88 ± 0.22; 0.66 ± 0.20
Zeta potential [mV]	–	−12.1 ± 1.8	−22.0 ± 0.93	−59.0 ± 5.1	−17.8 ± 8.0	<−30	0.155 ± 0.210; 0.16 ± 0.21
Encapsulation efficiency [%]	–	92.4 ± 5.2	97.3 ± 7.1	93.1 ± 5.0	88.7 ± 4.9	>90	–

## Data Availability

The data that support the findings of this study are available from the corresponding author upon reasonable request.
