# Supplementary material for: Self-Assembled Verteporfin Nanoparticles for Photodynamic and Light-Independent Therapy in Glioblastoma
Source: Adv Nanobiomed Res. Author manuscript; Available in PMC 2026 Jan 16. (PMC12806188; doi:10.1002/anbr.202400098)
Supplement: Suppl [file NIHMS2098697-supplement-Suppl.pdf]

Pump Rate	Drops per 100 µL	Estimated Drop Volume (µL)
2 mL/min	11.0 ± 0.8	9.1 ± 0.7
1 mL/min	11.3 ± 0.5	8.8 ± 0.4
0.5 mL/min	11.0 ± 0.8	9.1 ± 0.7
0.1 mL/min	11.3 ± 0.5	8.8 ± 0.4
0.01 mL/min	11.0 ± 0.8	9.1 ± 0.7
Handmade	12.7 ± 0.5	7.9 ± 0.3

Supplementary Table 1. Drops per 100 µL and estimated drop volume for each pump made NanoVP rate and handmade NanoVP. For both pump-made and handmade samples, NanoVP in DMSO accumulated at the tip of the needle or pipette and fell into the water under the force of gravity. There were no significant differences in the number of droplets per formulation method or the volume of each droplet. Data presented as mean ± standard deviation.

mg/kg NanoVP Dose, IP	Serum	Tumor-Bearing Hemisphere	Non-Tumor-Bearing Hemisphere
**0**	^^	^^	^^
**5**	3.0 ± 1.1	0.88 ± 0.25	0.13 ± 0.031
**10**	2.7 ± 1.1	0.71 ± 0.52	0.15 ± 0.15
**25**	5.6 ± 1.3	1.5 ± 0.36	0.33 ± 0.18

Supplementary Table 2. VP levels (µg/mL) in the serum and brain of mice with large, orthotopic GBM39 tumors in the right hemisphere two hours after intraperitoneal administration of NanoVP (0-25 mg/kg) as determined by HPLC-MS/MS. ^^ indicates below the quantifiable limit of detection. For 0 mg/kg, n=3 mice/group; for 5, 10, and 25 mg/kg, n=4 mice/group.

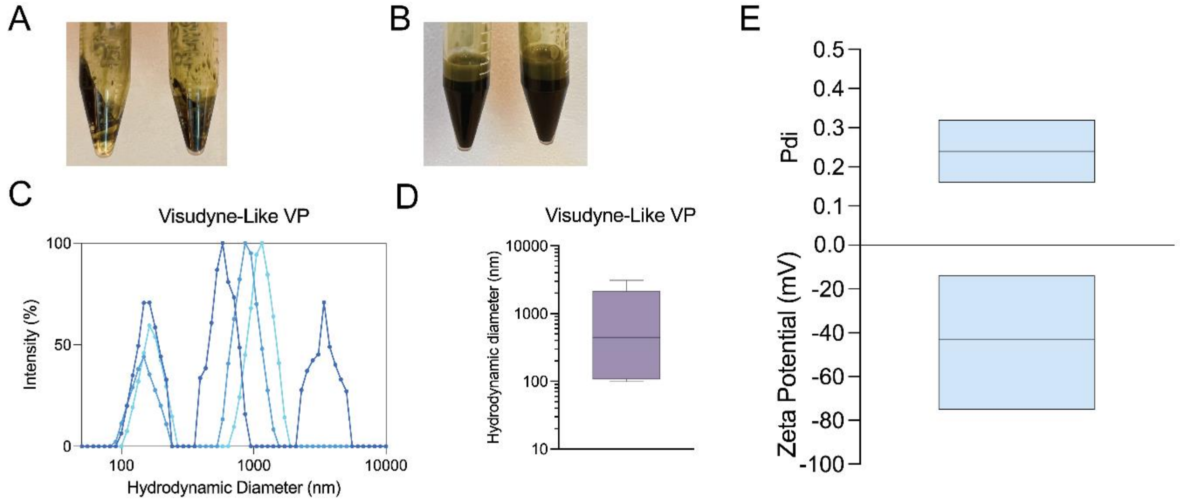

Supplementary Figure 1. Visudyne-like VP. (A) VP was mixed with appropriate lipids in chloroform, then flash-frozen in liquid nitrogen. The frozen mixture was lyophilized for 24 hours, then stored at -20°C in the dark until use. At the time of use, (B) the film was resuspended in PBS with calcium and magnesium via vortexing. Particle analysis revealed (C) broad peaks, indicating many particles of varying size. (D) The mean diameter was 1010 ± 1210 nm. (E) The Pdi was 0.24 ± 0.07, and the Zeta potential was -43.2 ± 25. DLS traces show three replicate reads from a single representative sample. Hydrodynamic diameter is presented with a bar at the mean, bars and whiskers extending to maximum and minimum, and the box extends from the lower to upper quartile. For Pdi and Zeta potential, a bar is at the mean, and boxes extend to the maximum and minimum.
